# Women-Doctors

**Published:** 1867-09

**Authors:** 


### *Women-Doctors.*

The following note explains itself. At the same time, we acknowledge the receipt of a Boston circular, to a similar effect:

"To THE EDITOR,

"*Dear Sir:*—Will you kindly insert the following in your paper, and aid a worthy cause?

"The New York Medical College for Women will begin their Fifth Annual Term, of 20 weeks, at the College in 12th Street, two doors east of Fourth Avenue, the first Monday in November. Address the Dean, Mrs. C. S. Lozier, M.D., 361 West

34th Street, N.Y., or the Secretary, Mrs. C. F. Wells, care of Fowler & Wells, N.Y."

It is in vain to protest against this thing, absurd as it clearly is. In this age, the greater the absurdity, the more strenuous its defence. The JOURNAL waives discussion.

***To Subscribers.***

Bills have been sent, as per published rates, to all subscribers who have not paid in advance for the JOURNAL. Mistakes will be cheerfully rectified, when pointed out, and full faith always given to the statements of our patrons. The books have been placed in the hands of a thoroughly competent bookkeeper, and order is rising from the chaos in which the present editor found affairs on remounting the tripod. Upon the conclusion of the *Endoscope*, space will be found in each number for a careful digest of current medical news, abstracts, items, etc. Lack of space has temporarily prevented the insertion of this chapter, which, hereafter, we hope to make one of large interest to our readers.

